# HIV-1 subtype diversity and transmission strain source among men who have sex with men in Guangxi, China

**DOI:** 10.1038/s41598-021-87745-3

**Published:** 2021-04-15

**Authors:** Yi Chen, Zhiyong Shen, Yi Feng, Yuhua Ruan, Jianjun Li, Shuai Tang, Kailing Tang, Shujia Liang, Xianwu Pang, Edward B. McNeil, Hui Xing, Virasakdi Chongsuvivatwong, Mei Lin, Guanghua Lan

**Affiliations:** 1 Institute of HIV/AIDS Prevention and Control, Guangxi Center of Disease Control and Prevention, Nanning, 530028 China; 2 State Key Laboratory of Infectious Disease Prevention and Control (SKLID), Chinese Center for Disease Control and Prevention (China CDC), Collaborative Innovation Center for Diagnosis and Treatment of Infectious Diseases, Beijing, 102206 China; 3 Epidemiology Unit, Faculty of Medicine, Prince of Songkla University, Hat Yai, 90110 Thailand

**Keywords:** Genetics, Molecular medicine

## Abstract

With the rapid increase in HIV prevalence of men who have sex with men (MSM) in recent years and common human migration and travelling across different provinces in China, MSM are now finding it easier to meet each other, which might contribute to local HIV epidemics as well as fueling cross-province transmission. We performed a cross-sectional survey in 2018–2019 to investigate the current HIV subtype diversity and inferred HIV strain transmission origin among MSM in Guangxi province, China based on a phylogenetic analysis. Based on 238 samples, we found that the HIV-1 subtype diversity was more complicated than before, except for three major HIV subtypes/circulating recombinant forms (CRFs): CRF07_BC, CRF01_AE, CRF55_01B, five other subtypes/CRFs (CRF59_01B, B, CRF08_BC, CRF67_01B, CRF68_01B) and five unique recombinant forms (URFs) were detected. In total, 76.8% (169/220) of samples were infected with HIV from local circulating strains, while others originated from other provinces, predominantly Guangdong and Shanghai. The high diversity of HIV recombinants and complicated HIV transmission sources in Guangxi MSM indicates that there has been an active sexual network between HIV positive MSM both within and outside Guangxi without any effective prevention. Inter-province collaboration must be enforced to provide tailored HIV prevention and control services to MSM in China.

## Introduction

Phylogenetics can provide an insight into the spread of infectious diseases such as HIV. It is known that even though HIV continually evolves and adapts in infected hosts, there are stringent bottlenecks leading to transmission of a single monophyletic variant to most (> 95%) newly-infected persons^1^. Hence, information about the extent of genetic diversity at the population level can be attained by reconstructing HIV phylogenetic trees, including where different viral strains might have originated, and whether new strains are entering populations as an epidemic proceeds^2^. When combining HIV phylogenetic analysis with epidemiological information, HIV epidemiology can be classified into three general categories: (1) molecular Epidemiology, which allows an understanding of the risk factors for HIV transmission and epidemic spread; (2) phylodynamics, which reconstructs the epidemic history and quantifies epidemic growth or decline using viral genealogies and explicit population genetic models; and (3) phylogeography, which describes the distribution of subtype diversity, estimates the impact of human migration on viral spread, and places historical and risk factor data into geographic context in order to identify transmission hubs^3^.

It is well known that phylogenetic analyses have elucidated the origin and spread of the pandemic human immunodeficiency virus type (HIV-1) group M virus, both during the pre-epidemic period and the epidemic phase of spread among humans^4^. Additionally, it is also applied to evaluate small transmission chains between infected individuals, such as some forensic studies^5–7^, and to track the founder events that resulted in the spread of HIV-1 strains across vast geographic areas, specific countries, and within geographically restricted communities^8^.

Guangxi Zhuang Autonomous Region (Guangxi) is located in southwest China bordering Vietnam. The HIV epidemic in Guangxi has been driven by injecting drug users since 1996 and shifted to heterosexual transmission from 2006 onwards^9^. Meanwhile, transmission among men who have sex with men (MSM) has also increased and contributed to the HIV epidemic in Guangxi. The first MSM HIV case was reported in 2005 and since then, the number of reported HIV MSM cases has increased from 45 in 2008 to 868 in 2018. Annual surveillance data indicated that the HIV prevalence increased from 0.83% in 2008 to 5.0% in 2012, rose sharply to 11.2% in 2015^10^, and stabilized at around 10% from 2017 to 2018.

A study conducted in Guangxi in 2013 targeting newly diagnosed HIV positive cases aged 18 years or older identified four circulating recombinant forms (CRFs) among MSM cases comprising CRF07_BC (44.4%), CRF01_AE (38.9%), CRF55_01B (11.1%) and CRF08_BC (5.6%)^11^. However, in recent years, with the development of modern transportation, especially the establishment of high speed rail, MSM traveling to seek sexual partners outside of their local residence has become more common. Gay websites and Gay mobile phone applications are popular mediums for Chinese MSM seeking male sexual partners^12, 13^. In order to understand the changing situation of the HIV epidemic in China, information of HIV cross-province transmission among MSM should be documented. The objective of our study is to identify the current diversity of HIV subtypes in Guangxi MSM, and further analyze the temporal-geographical origins of HIV transmission sources.

## Methods

### Study participants and blood collection

Our study participants were recruited from an antiviral therapy (ART) clinic and a MSM Voluntary Counseling and Testing (VCT) clinic in Nanning from September 2018 to November 2019. These were subjects who had started attending the clinics from 2007 to 2019. In 2018, we recruited 371 subjects, 328 of whom were already on ART, of which HIV DNA was successfully amplified in 192. In 2019, we screened 52 treatment-naïve subjects from a VCT clinic, of which 46 gave HIV RNA amplification. Finally, 238 HIV nucleotide sequences were obtained for genotyping and phylogenetic analysis (see Supplementary Fig. S1 for details). Socio-demographic data comprising age, marital status, area of residence, ethnicity, sexual identity and education level were collected based on face-to-face structured interviews by trained research staff.

### Ethics statement

The study was approved by the Guangxi institutional review board (approval number GXIRB 2019-0010-1). Informed consent from all participants was obtained after full explanation of the contents was given. All participants in this study were de-identified to maintain their anonymity. All research methods in this study were performed in accordance with the approved guidelines.

### HIV DNA/RNA extraction, amplification, and sequencing

Whole blood specimens were collected and plasma was isolated and sent under cold chain to the laboratory in our study. Since the patients on antiretroviral treatment were virally suppressed, HIV DNA from subjects on ART were extracted from 200 μl of whole blood using the QIAamp DNA Blood Mini Kit (250) according to the manufacturer’s handbook. HIV RNA from ART-naïve subjects was extracted from 200 μl of plasma by the NucliSENS easyMAG platform (BioMérieux, Boxtel, Netherlands) as per the manufacturer’s instructions. Then, nested polymerase chain reaction (PCR) was used for viral DNA /RNA to obtain fragments of the pol gene (HXB2, positions 2147–3462 for a total of 1315 bp)^14^. Primers MAW26 (5′-TGGAAATGTGGAAAGGAAGGAC-3′ and RT21 (5′-CTGTATTTCTGCTATTAAGTCTTTTGATGGG-3′) in 25 μl reaction volumes was used for pol fragment first amplified with One Step Reverse Transcription PCR (Takara, Dalian, China) and Cycling conditions were as follows: 50 °C for 30 min; 94 °C for 2 min; 30 cycles of 94 °C for 30 s, 55 °C for 30 s, and 72 °C for 2 min 30 s; and 72 °C for 10 min. Then, the second PCR was conducted using 2 × Taq PCR MasterMix (Tiangen, Beijing, China) with primers Pro-1 (5′-CAGAGCCAACAGCCCCACCA-3′) and RT-20 (5′-CTGCCAGTTCTAGCTCTGCTTC-3′) in 50 μl reaction volumes. The following cycling conditions were applied: 94 °C for 5 min; 30 cycles of 94 °C for 30 s, 63 °C for 30 s, and 72 °C for 2 min 30 s; and 72 °C for 10 min. Each step was operated with appropriate negative controls to detect possible contamination throughout the experiments. PCR products were analyzed with 1% agarose gel electrophoresis. Positive PCR products were purified using QIAquick Gel Extraction Kit (Qiagen, Valencia, CA, USA) and sequenced directly on an ABI 3730XL automated sequencer with BigDye terminators (Applied Biosystems, Foster City, CA, USA) by Beijing Biomed Technology Development Co., Ltd (Beijing, China).

### HIV subtyping

HIV subtypes were determined based on Maximum-likelihood tree analysis, which was generated using IQ TREE 2.0.6^15^ with the best-fitting model of TVM + F + R5. This software can determine best-fitting model for the aligned study sequences automatically^16, 17^. The topology of trees was tested by ultrafast bootstrap (UFBoot) approximation with 1000 bootstrap replicates to reduce computing time while achieving more unbiased branch supports^18^. Clusters with a bootstrap value ≥ 0.95 (95%) were defined as a phylogenetic cluster^17^.

248 reference sequences were used for HIV subtype determination. 170 HIV-1 reference sequences of HIV-1 M,N,O,P group were downloaded from Los Alamos National Laboratory (LANL) HIV Sequence Database (https://www.hiv.lanl.gov/content/sequence/NEWALIGN/align.html, accessed in July, 2020) by BLAST (see Supplementary S1). 59 CRF reference sequences were obtained by BLAST from China local HIV sequence dataset from the Division of Virology and Immunology, National Center for AIDS/STD Control and Prevention (NCAIDS), China CDC representing the common circulating strains in China, including subtypes of CRF01_AE, CRF07_BC, CRF08_BC, CRF55_01B and CRF59_01B. Alignment was done using the “HIV ALIGN” software on the LANL HIV Sequence Database, and manual adjustments were followed using BioEdit software^19^, considering protein coding sequences. Based on the initial Neighbor-joining tree (Reconstructed by MEGA 7.0), most of the subtypes of sample sequences were found to be close to CRF01_AE, CRF07_BC, CRF55_01B, CRF59_01B. Therefore, HIV BLAST was performed on HIV sequence database for some sample sequences close to these four subtypes, and the reference sequences achieved were added into the previous reference sequences dataset. Finally, after removal of duplicated sequences, 248 reference sequences for our 238 study sample sequences were obtained, containing 81 CRF01_AE, 70 CRF07_BC, 7 CRF55_01B, 7 CRF5901_B and 83 other subtype reference sequences.

### HIV genetic network construction

The genetic network was constructed using HIV-TRACE (Transmission Cluster Engine, http://demo.hivtrace.org/network.html)^20^. Sample HIV pol sequences were aligned to HXB2 reference sequence and the pairwise genetic distances were calculated under the Tamura-Nei 93 model^21^. 8 sequences with ambiguous nucleotides were removed. The genetic distance between nodes (each node represented a sequence or an individual) up to 0.15% substitutions per site were linked to each other. The genetic distance threshold of 0.15% was recommended by the Centers for Disease Control and Prevention (CDC) in the United States (US)^22^.

### HIV transmission strain source inference

HIV transmission strain sources were analyzed using a Bayesian temporal-geographical origin analysis. As there were only four sequences with CRF59_01B, three sequences with subtype B, two sequences for CRF_08BC and one for CRF67_01B, CRF68_01B, CRF01_BC respectively, no phylogenetic trees were needed to determine their transmission source. We determined the transmission source of the strains among HIV positive MSM individuals with CRF01_AE, CRF07_BC, and CRF55_01B, respectively.

Regarding the pol gene (PR-RT region) reference sequences selection, which was the second reference dataset in our study, the five most similar sequences to each sample sequence from the Los Alamos HIV Sequence Database were selected using the “HIV BLAST” tool (accessed June, 2020). 1,100 reference sequences were obtained and duplicated sequences and those without sampling year or geographical information were removed. Finally, 374 unique pol sequences with sampling time and geographical information were obtained and used as reference sequences for determining HIV transmission strain sources for our 220 pol gene sample sequences. Among the 374 reference sequences, one was from Vietnam and the rest were from China. The 594 (374 + 220) pol sequences were separated into three datasets by different subtypes, CRF01_AE (n = 299), CRF07_BC (n = 243), and CRF55_01B (n = 52). These three sequence datasets were aligned by “HIV ALIGN” on the Los Alamos HIV Sequence Database (accessed June, 2020).

To reconstruct the spatial dynamics and estimate the sources of the strains of our study participants, a Bayesian discrete phylogeographic approach was performed using Markov chain Monte Carlo (MCMC) runs of 300 million generations with BEAST v.1.8.4 under a Bayesian skygrid demographic model. The first 10–30% of the states from each run were discarded as the burn-in^23, 24^. All three data sets were analyzed using a general time-reversible (GTR) model specifying a gamma distribution based on a Neighbor-Joining tree as a prior on each relative substitution rate and a relaxed uncorrelated lognormal (UCLN) molecular clock model to infer the timescale of HIV evolution with a gamma distribution prior on the mean clock rate (shape = 0.001, scale = 1000)^25^. The Bayesian MCMC output was analyzed using Tracer v1.6 (http://beast.bio.ed.ac.uk/Tracer). The Maximum clade credibility (MCC) trees were generated in Tree Annotator v1.8.4. Finally, the most probable temporal and geographical origin of HIV transmission strains for the study participants were inferred according to the output of the posterior of Bayesian estimation and visualized on MCC trees using the FigTree software v1.4.3 (http://beast.bio.ed.ac.uk). The tree figure editing was using Adobe Illustrator 2020(V 24.0.1.341).

Sequences with posterior probability over 0.7 were defined as Guangxi local circulating strains, and the posterior probability refers to the support of locations at the nodes. The HIV transmission strain source provinces of our study samples were then tabulated by different HIV subtypes.

### Statistical analysis

Statistical analyses for this study were performed using the R language and environment (R version 4.0.0). The distribution of HIV subtypes with epidemiological characteristics were described. Chi-squared tests were used to test the associations between HIV subtypes and epidemiological characteristics, as well as the association between HIV subtype and HIV transmission source.

### Study nucleotide sequence

Considering the risk of breaching patient confidentiality, we submitted a random sample of 25% of all HIV pol gene nucleotide sequences obtained from our study to GenBank under accession numbers MW573881- MW573938. (see Supplementary S2 for details).

## Results

### Demographic characteristics of study samples

Table 1 summarizes demographic information of the 238 study participants. The sample was predominated by young adults, single, rural residents, Han ethnicity, homosexual sexual identity, and a college or higher education level. Three major HIV subtypes were identified; CRF01_AE, CRF07_BC, and CRF55_01B with a small proportion of others. The only variable associated with HIV subtypes was area of residence; except for the CRF01_AE subtype, a higher proportion of participants with other subtypes lived in a rural area.

**Table 1:** Demographic characteristics for different HIV-1 genotypes among MSM in Nanning.

Variable	CRF01_AE	CRF07_BC	CRF55_01B	Others	Total	P value (χ^2^ test)
Total	89	107	24	18	238 (100.0)	
**Age group (years)**						
≤ 25	34 (40.0)	43 (43.0)	8 (36.4)	5 (29.4)	90 (40.2)	0.878
26–35	34 (40.0)	41 (41.0)	9 (40.9)	7 (41.2)	91 (40.6)	
> 36	17 (20.0)	16 (16.0)	5 (22.7)	5 (29.4)	43 (19.2)	
**Marital status**						
Married	9 (10.3)	11 (10.4)	3 (12.5)	5 (27.8)	28 (11.9)	0.214
Single	78 (89.7)	95 (89.6)	21 (87.5)	13 (72.2)	207 (88.1)	
**Residence**						
Rural	38 (43.7)	69 (65.7)	15 (62.5)	10 (55.6)	132 (56.4)	0.020
Urban	49 (56.3)	36 (34.3)	9 (37.5)	8 (44.4)	102 (43.6)	
**Ethnicity**						
Han	50 (61.7)	62 (62.0)	12 (54.5)	11 (61.1)	135 (61.1)	0.931
Zhuang	31 (38.3)	38 (38.0)	10 (45.5)	7 (38.9)	86 (38.9)	
**Sexual identity**						
Bisexual	16 (19.5)	19 (19.6)	4 (19.0)	4 (22.2)	43 (19.7)	0.984
Homosexual	66 (80.5)	78 (80.4)	17 (81.0)	14 (77.8)	175 (80.3)	
**Education**						
Junior school or less	9 (10.3)	13 (12.4)	4 (17.4)	6 (33.3)	32 (13.7)	0.178
High school	32 (36.8)	38 (36.2)	10 (43.5)	7 (38.9)	87 (37.3)	
College or higher	46 (52.9)	54 (51.4)	9 (39.1)	5 (27.8)	114 (48.9)	
**HIV diagnosed year**						
2007–2016	40 (44.9)	48 (44.9)	7 (29.2)	5 (27.8)	100 (42.0)	0.285
2017–2019	49 (55.1)	59 (55.1)	17 (70.8)	13 (72.2)	138 (58.0)	

Column totals may not necessarily add up due to missing values.

### HIV subtypes among MSM in Guangxi

**Figure 1:**
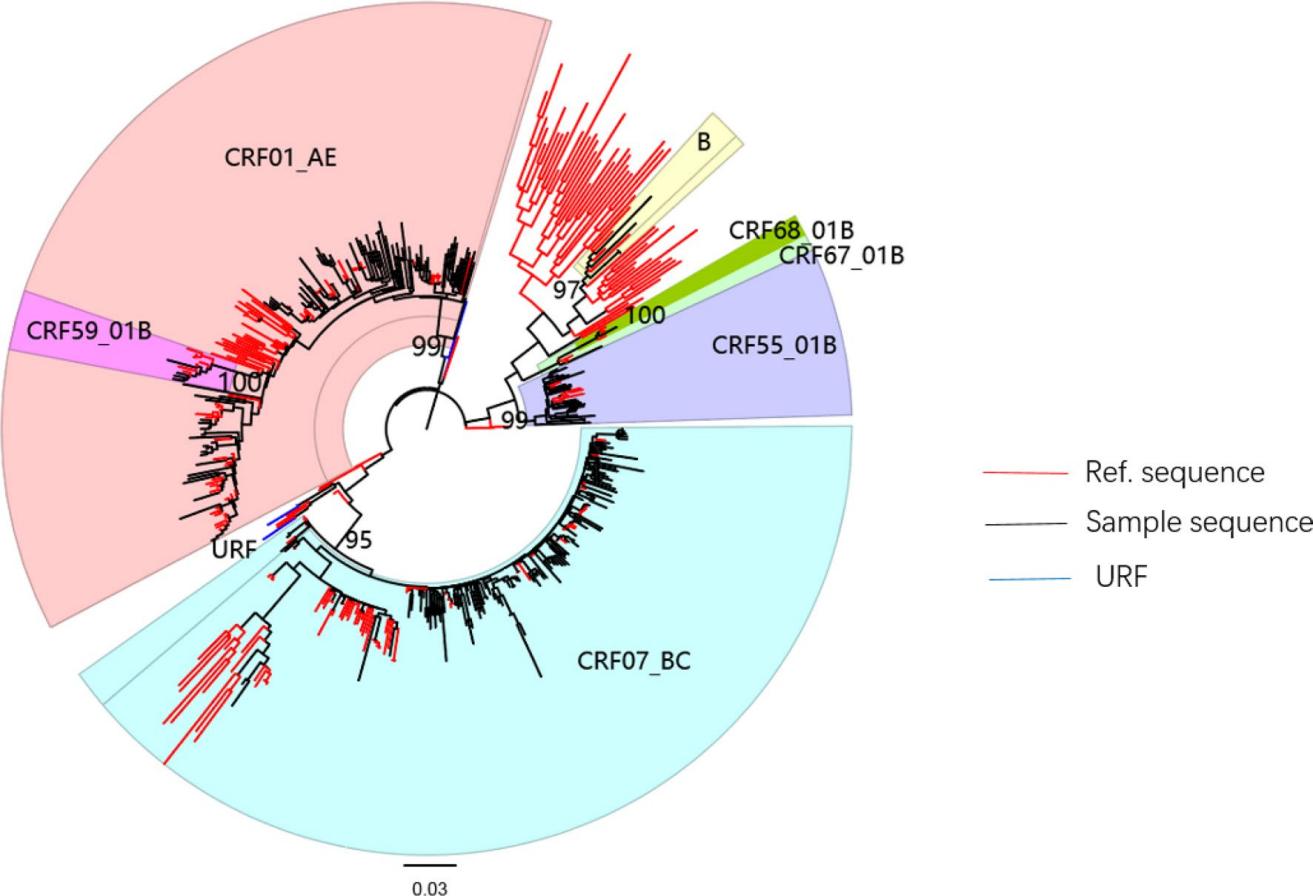
Maximum-likelihood phylogenetic trees of HIV-1 pol sequences from 238 MSM in Nanning, Guangxi, China. The topology of trees was tested by IQ Tree (V.2.0.6^15^)ultrafast bootstrap (UFBoot) approximation with 1000 bootstrap replicates, the bootstrap values were showed on the corresponding nodes, Clusters with bootstrap value ≧0.95 (95%) were defined as phylogenetic cluster. Red clade denoted reference sequence, black clade denoted samples sequences and blue clade represented URF.

Figure 1 illustrates the maximum-likelihood phylogenetic tree based on sample and reference sequences. References with a red clade represents those commonly identified around the world. The samples were predominated by three major HIV CRFs: CRF07_BC, CRF01_AE, CRF55_01B. Five other subtypes/CRFs were CRF59_01B, B, CRF08_BC, CRF67_01B, and CRF68_01B. Five unique recombinant forms (URFs) were discovered (shown with a blue clade in Fig. 1).

## HIV genetic network construction

Under the genetic distance threshold of 0.15%, 52.6% (121/230) of nodes (sequences) were included into the genetic network, 25 clusters were identified, and the largest cluster consisted of 38.8% (47/121) of nodes, which had subtype CRF07_BC. The sizes of the remaining clusters ranged from 2 to 7. This indicates that sequences with CRF07_BC tended to gather as one big cluster than other subtypes. The result is shown in Supplementary Figure S2.

### HIV transmission strain sources

Subtypes/CRF B, CRF59_01B, CRF08_BC, CRF67_01B and CRF68_01B were excluded from the analysis due to small sample sizes (n < 5).

The HIV transmission strain sources for 220 sample sequences with CRF01_AE, CRF07_BC, CRF55_01B were visualized on MCC trees (see Supplementary Fig. S3 for details). Based on the MCC trees, most Guangxi local circulating strains were spread directly from Guangdong province. There were two clusters of HIV CRF01_AE strains originating from Guangdong province and then spreading directly into Guangxi, or via Shanghai/Liaoning province into Guangxi. HIV CRF07_BC strains were spread into Guangxi by three clusters, and most Guangxi local strains were spread directly from Guangdong, followed by originating in Guangdong province and then spreading through Shanghai into Guangxi. While there were two clusters spreading HIV CRF55_01B strains from Guangdong to Guangxi directly, these developed into Guangxi local circulating strains. The temporal signal was indicated by the Bayesian phylogeographic analysis results. The probable time of introduction for CRF01_AE strains with effective population size circulating in Guangxi MSM was probably in 2006.2 (2004.3-2008.4) from Liaoning province but originated from Guangdong, for CRF07_BC strains was probably in 2008.6 (2006.9-2010.5) from Guangdong, and CRF55_01B was probably in 2008.02 (2007.5–2008.6) from Guangdong. The temporal signal was indicated by a red dot on the Bayesian MCC trees for three subtypes and shown in Supplementary Fig. S3.

The source provinces were summarized by three common subtypes and groups of participants in Tables 2 and 3. Based on the criteria of posterior probability over 0.7, transmission source of 196 samples (89.1%) could be determined. The HIV transmission source of 169 samples (76.8%) were Guangxi local circulating strains. The remaining 27 strains were from outside Guangxi. 101 (96.2%) of 105 HIV strains with CRF07_BC were primary local strains in Guangxi MSM community, compared to 50 (74.6%) of 67 CRF01_AE and 18 (75.0%) of 24 CRF55_01B (Fisher’s exact test, *p* < 0.001). The subtype with the most diversified source was CRF01_AE. Subjects from ART clinic and those from VCT clinic did not differ in source of their HIV strains (Fisher’s exact test, *p* = 0.288).

**Table 2:** Distribution of HIV transmission strains circulating provinces.

Posterior probability	Province	Total	Subtype			Sample source	
			CRF01_AE	CRF07_BC	CRF55_01B	ART clinic	VCT clinic
> 0.7	Guangxi	169	50	101	18	140	29
	Guangdong	17	10	1	6	16	1
	Shanghai	6	3	3	0	1	5
	Beijing	1	1	0	0	1	0
	Shanxi	2	2	0	0	1	1
	Liaoning	1	1	0	0	1	0
< 0.7		24	22	2	0	20	4
Total		220	89	107	24	180	40

**Table 3:** Comparison of HIV transmission source by different subtype and sample group.

Province	Total	Subtype				Sample source		
		CRF01_AE	CRF07_BC	CRF55_01B	*P* (Fisher’s Exact test)	ART clinic	VCT clinic	*P* (Fisher’s Exact test)
Guangxi	169	50	101	18	< 0.001	140	29	0.288
Non-Guangxi	27	17	4	6		20	7	

## Discussion

In this study we found that the majority of HIV positive MSM in Guangxi were young, single, identified themselves as homosexual, and were well educated. The proportion aged 25 years or less was higher than those in studies from Shenzhen, Beijing and Shanghai^26–28^, as well as the United States^29^. Our sample was similar to those in Kunming^30^ and Thailand^31^.

The three major CRFs identified were CRF07_BC, CRF01_AE and CRF55_01B. Additionally, five CRFs (four of which were newly detected in Guangxi in recent five years) and five URFs were detected. We created a table (see Supplementary Table S1) to compare the HIV subtype/CRF diversity in western and Asian countries, as well as provinces or large cities in China. The HIV subtype/CRF diversity in Guangxi MSM in the current study was more complicated than that in 2013^11^ and those in southeast countries such as Thailand^31^, Malaysia^32^ and Singapore^33^. Degrees of diversity were high in Shanghai^28^, Guangdong^26^, Beijing^34^ and Sichuan^35^. The predominant CRFs in our study were similar to other provinces in China^26, 35, 36^, but different from those in southeast Asian countries^31–33^, United States^29^, Canada and European countries^37–41^. The newly detected CRFs in our study had been circulating in the northeast and middle and southern parts of China since 2013^42–46^, which concurred with the results from our temporal geographical analysis.

In our study, genetic network analysis identified one large cluster with subtype CRF07_BC, while the Bayesian phylogeographic analysis confirmed that the majority of Guangxi local HIV circulating strains were CRF07_BC. This subtype increased rapidly in recent years and has been seen among MSM in many cities of China. Among Shenzhen MSM, CRF07_BC increased from 12.5% in 2006 to 43.2% in 2012^26^. In Fujian, the proportion of CRF07_BC infections among recently infected MSM expanded rapidly from 19.0% in 2012 to 41.9% in 2013^47^. A phylodynamic analysis also revealed that the CRF07_BC strain had driven two rapid HIV spreading waves with effective population size in China, the second wave coincided with the expanding MSM cluster^48^. HIV CRF01_AE, the second-most common subtype in our study, originated in Thailand in the early 1990s^49, 50^. This subtype had been circulating among IDUs and sexually active populations in China for over 30 years^51, 52^. It is relatively virulent in that it causes rapid CD4 T-cell decline and rapid disease progression^53^. This may explain its ineffective transmission and reported decline since 2010.

More than 86% of HIV infections from our sample originated from Guangxi local circulating strains. For strains circulating outside Guangxi, all except four were probably transmitted from economic developed cities of China, such as Guangdong and Shanghai. The most probable time of the three common HIV CRF strains were effectively introduced into Guangxi MSM populations between 2006 and 2008.

The source provinces of HIV strains were not neighboring provinces of Guangxi with low economic level such as Yunnan and Guizhou. In fact, most were from large cities of China including Guangdong and Shanghai. They contributed to a new emergence of CRFs and URFs of HIV and increased the complexity of the HIV epidemic among MSM populations in Guangxi. Previous studies reported that MSM living in south-western rural China tended to migrate to the eastern urban areas of China^54, 55^ where the HIV prevalence is high^56–58^. This migration might have facilitated HIV transmission back to the provinces where these MSM came from. Although a large proportion of MSM tended to be infected from local circulating strains, the proportion of MSM infected with HIV from non-local circulating strains (13.7%) is still higher than that of Guangxi heterosexuals (5.9%)^11^ which suggests that the MSM sexual network is broader and more complicated than the heterosexual one.

Our study has certain limitations. First, the samples were collected from an ART clinic and a VCT clinic. Although we could get DNA sequences from ART clinic subjects, we could not get RNA sequences because their virus had been well suppressed. However, this has little effect on the HIV strain transmission source inference or genetic network study^59^. On the other hand, the subjects from the VCT clinic might not well represent recently infected MSM, most of whom might not have access to this service.

In summary, a high diversity of HIV recombinants and complicated HIV transmission sources were found in Guangxi MSM. This suggests that there has been an active sexual network between HIV positive MSM both within and outside Guangxi without effective prevention. Inter-province collaboration must be enforced to provide tailored HIV prevention and control services to MSM in China.

## Supplementary Information

Supplementary information.
