# Supplementary material for: HIV-1 subtype diversity and transmission strain source among men who have sex with men in Guangxi, China
Source: Sci Rep. 2021 Apr 15;11:8319. doi: 10.1038/s41598-021-87745-3 (PMC8050077; doi:10.1038/s41598-021-87745-3)
Supplement: Supplementary file 1 — Supplementary information. [file 41598_2021_87745_MOESM1_ESM.pdf]

Supplementary

HIV-1 Subtype Diversity and Transmission Strain Source among Men Who Have Sex with Men in Guangxi, China

Yi Chen[1], Zhiyong Shen[1], Yi Feng[1, 2], Yuhua Ruan[1, 2], Jianjun Li[1], Shuai Tang[1,+], Kailing Tang[1,+], Shujia Liang[1,+], Xianwu Pang[1,+], Edward B McNeil[3], Hui Xing[1, 2],Virasakdi Chongsuvivatwong[3,*] Mei Lin[1,*] and Guanghua Lan[1,*].

1 Institute of HIV/AIDS Prevention and Control, Guangxi Center of Disease Control and Prevention, Nanning, 530028, China

2 State Key Laboratory of Infectious Disease Prevention and Control (SKLID), Chinese Center for Disease Control and Prevention (China CDC), Collaborative Innovation Center for Diagnosis and Treatment of Infectious Diseases, Beijing, 102206, China.

3 Epidemiology Unit, Faculty of Medicine, Prince of Songkla University, Hat Yai, 90110, Thailand.

* cvirasak@medicine.psu.ac.th , gxlinmei@126.com and lgh605@163.com.

+ These authors contributed equally to this work

S1. Details of reference sequences downloaded from Los Alamos National Laboratory (LANL) HIV Sequence Database for HIV subtype determination

170 latest HIV-1 reference sequences of HIV-1 M,N,O,P group were downloaded from HIV sequence database(https://www.hiv.lanl.gov/content/sequence/NEWALIGN/align.html, accessed in July,2020), included A1(3 sequences),A2(3),B(4),C(4),D(4),F1(4),F2(4),G(4),H(4),J(3),K(2),CRF01_AE(3),CRF02_AG(3),CRF03_AB(1),CRF04_CPX(3),CRF05_DF(3),CRF06_CPX(3),CRF07_BC(3),CRF08_BC(2),CRF09_CPX(4),CRF10_CD(3),CRF11_CPX(3),CRF12_BF(3),CRF13_CPX(3),CRF14_BG(3),CRF15_01B(3),CRF16_A2D(2),CRF17_BF(3),CRF18_CPX(3),CRF19_CPX(3),CRF20_BG(1),CRF21_A2D(3),CRF22_01A1(2),CRF23_BG(2),CRF24_BG(3),CRF25_CPX(3),CRF26_AU(3),CRF27_CPX(2),CRF28_BF(3),CRF29_BF(3),CRF31_BC(3),CRF32_06A1(1),CRF33_01B(3),CRF34_01B(1),CRF35_AD(3),CRF36_CPX(2),CRF37_CPX(2),CRF38_BF1(3),CRF39_BF(3),CRF40_BF(3),CRF42_BF(1),CRF43_02G(3),CRF44_BF(1),CRF45_CPX(3),CRF46_BF(3),CRF47_BF(2),CRF49_CPX(3),O(4),N(3),P(2),CPZ(3).

S2 Genbank accession numbers

MW573881, MW573882, MW573883, MW573884, MW573885, MW573886, MW573887, MW573888, MW573889, MW573890, MW573891, MW573892, MW573893, MW573894, MW573895, MW573896,MW573897, MW573898, MW573899, MW573900, MW573901, MW573902, MW573903, MW573904, MW573905, MW573906, MW573907, MW573908, MW573909, MW573910, MW573911, MW573912, MW573913, MW573914, MW573915, MW573916, MW573917, MW573918, MW573919, MW573920, MW573921, MW573922, MW573923, MW573924, MW573925, MW573926, MW573927, MW573928, MW573929,

MW573930, MW573931, MW573932,MW573933,MW573934, MW573935, MW573936, MW573937, MW573938

Table S1 The diversity of HIV subtype/CRFs among MSM in different countries/regions/cities

ID	Location	Year	Predominant subtype/CRF	Others
1	Guangxi (Current study)	2018-2019	CRF01_AE, CRF07_BC, CRF55_01B	CRF08_BC, CRF59_01B, CRF67_01B,CRF68_01B and URFs
2	Guangxi[12]	2013	CRF01_AE, CRF07_BC, CRF55_01B	CRF08_BC
3	Guangdong(Shenzhen)[15]	2005-2012	CRF01_AE, CRF07_BC, CRF55_01B, B	C, CRF08_BC, CRF67_01B, CRF33_01B and URFs
4	Shanghai[17]	2009-2013	CRF01_AE, CRF07_BC,	B, CRF55_01B, CER67_01B, CRF68_01B, CRF08_BC, CRF59_01B and URFs
5	Beijing[23]	2019	CRF01_AE, CRF07_BC	B, CRF55_01B, CRF59_01B, CRF65_cpx, CRF79_0107, A, URFs (0107, 01B and 01BC)
6	Sichuan[24]	2011-2017	CRF01_AE, CRF07_BC	B, CRF08_BC, CRF52_01BC, CRF55_01B,C and URFs
7	Kunming[48]	2010-2012	CRF01_AE, CRF07_BC	B, CRF08_BC and URFs
8	Hong Kong[49]	1994-2013	CRF01_AE, B	CRF07_BC, C, CRF02_AG, CRF08_BC, A1, G, D, CRF06_cpx, F, CRF12_BF and URFs
9	Thailand[20]	2009-2015	CRF01_AE, B, CRF01_AE/B	C and URFs
10	Malaysia[21]	2006-2012	CRF01_AE, B	-
11	Singapore[22]	2008-2009	B, CRF01_AE, CRF51_01B	-
12	Nine European countries and Canada[26]	2014	B,C	A, CRF01_AE, CRF02_AG, D,G,F
13	United States[18]	2006-2013	B	C, CRF02_AG, A, CRF01_AE, and G.

Table S2. Demographic characteristics for different HIV-1 genotypes among MSM in Nanning.

**Variable**	**CRF01_AE**	**CRF07_BC**	**CRF55_01B**	**Total**	**P value (χ^2 test)**
Total	89(100.0)	107 (100.0)	24 (100.0)	220 (100.0)	
Age group (years)					0.924
≤ 25	34 (40.0)	43 (43.0)	8 (36.4)	85(41.1)	
26-35	34 (40.0)	41 (41.0)	9 (40.9)	84(40.6)	
>36	17 (20.0)	16 (16.0)	5 (22.7)	38(18.4)	
Marital status					0.95
Married	9 (10.3)	11 (10.4)	3 (12.5)	23(10.6)	
Single	78 (89.7)	95 (89.6)	21 (87.5)	194(89.4)	
Residence					0.007
Rural	38 (43.7)	69 (65.7)	15 (62.5)	122(56.5)	

Urban	49 (56.3)	36 (34.3)	9 (37.5)	94(43.5)	
Ethnicity					0.800
Han	50 (61.7)	62 (62.0)	12 (54.5)	124(61.1)	
Zhuang	31 (38.3)	38 (38.0)	10 (45.5)	79(38.9)	
Sexual identity					0.998
Bisexual	16 (19.5)	19 (19.6)	4 (19.0)	39(19.5)	
Homosexual	66 (80.5)	78 (80.4)	17 (81.0)	161(80.5)	
Education					0.784
Junior school or less	9 (10.3)	13 (12.4)	4 (17.4)	26(12.1)	
High school	32 (36.8)	38 (36.2)	10 (43.5)	80(37.2)	
College or higher	46 (52.9)	54 (51.4)	9 (39.1)	109(50.7)	
HIV diagnosed year					0.340
2007-2016	40 (44.9)	48 (44.9)	7 (29.2)	95(43.2)	
2017-2019	49 (55.1)	59 (55.1)	17 (70.8)	125(56.8)	

Note: Removal of samples with “subtype=other”

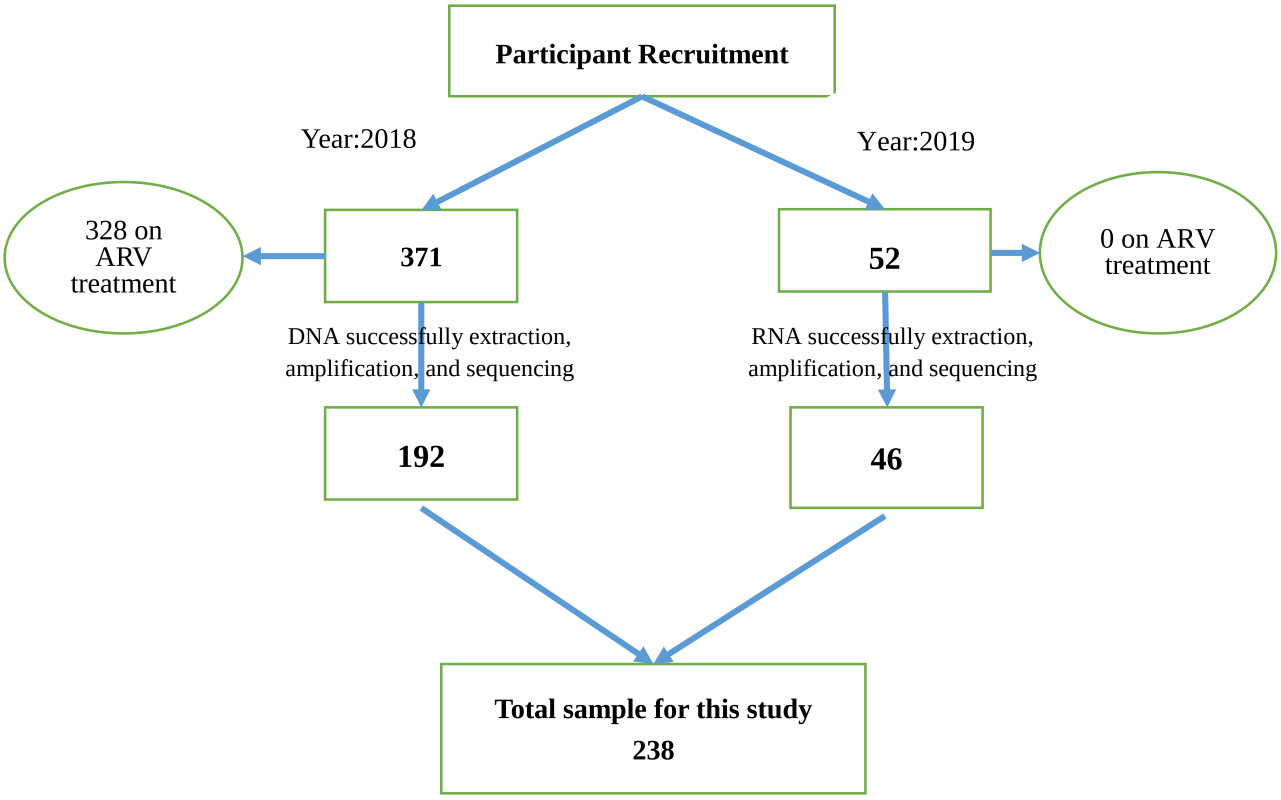

Figure S1 Flow chart of study participants’ recruitment

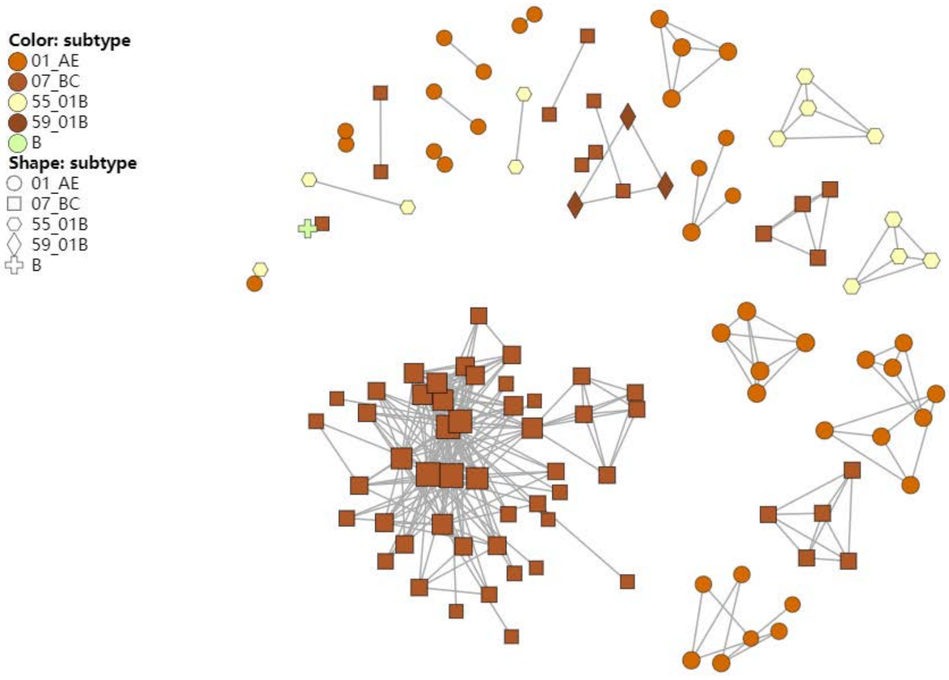

Figure S2 HIV transmission network for study samples specified by subtype (Genetic distance threshold=0.015). The subtypes of samples were indicated in the upper left, the figure was drawn by using HIV-TRACE (Transmission Cluster Engine, http://demo.hivtrace.org/network.html)[20].

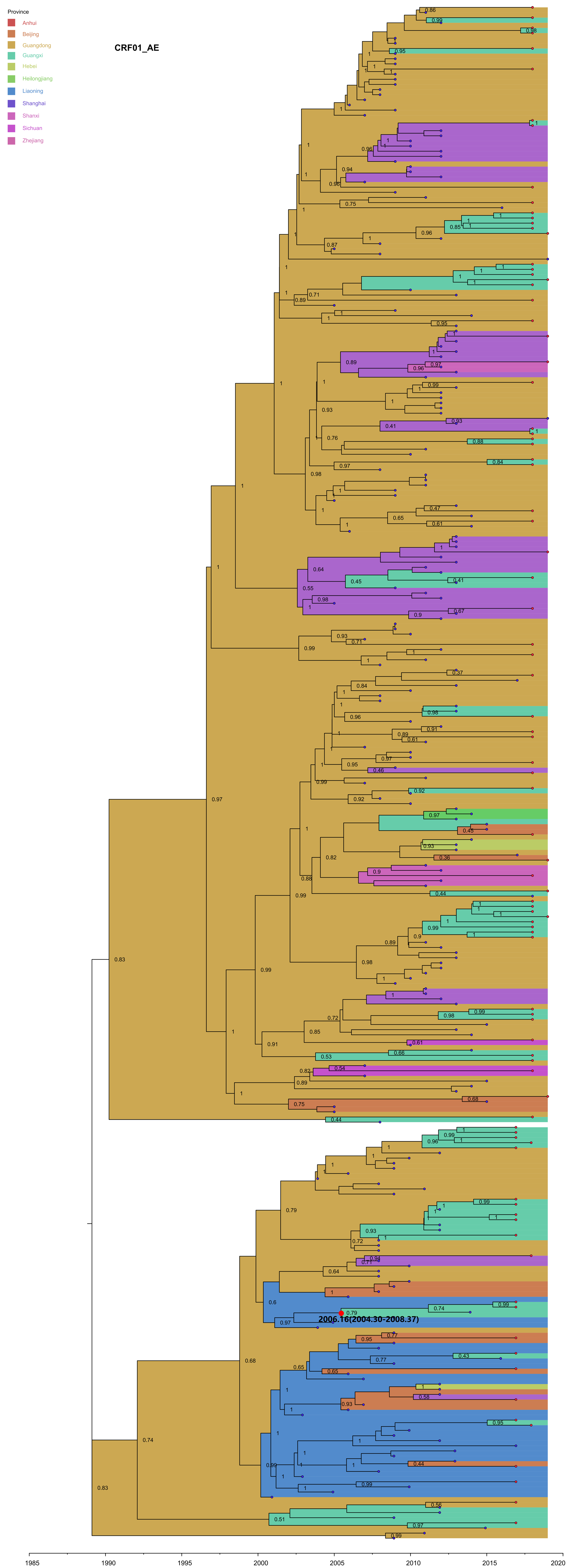
Province
Anhui
Beijing
Guangdong
Guangxi
Hebei
Heilongjiang
Liaoning
Shanghai
Shanxi
Sichuan
Zhejiang
CRF01_AE
2006.16(2004.30-2008.37)
1985
1990
1995
2000
2005
2010
2015
2020

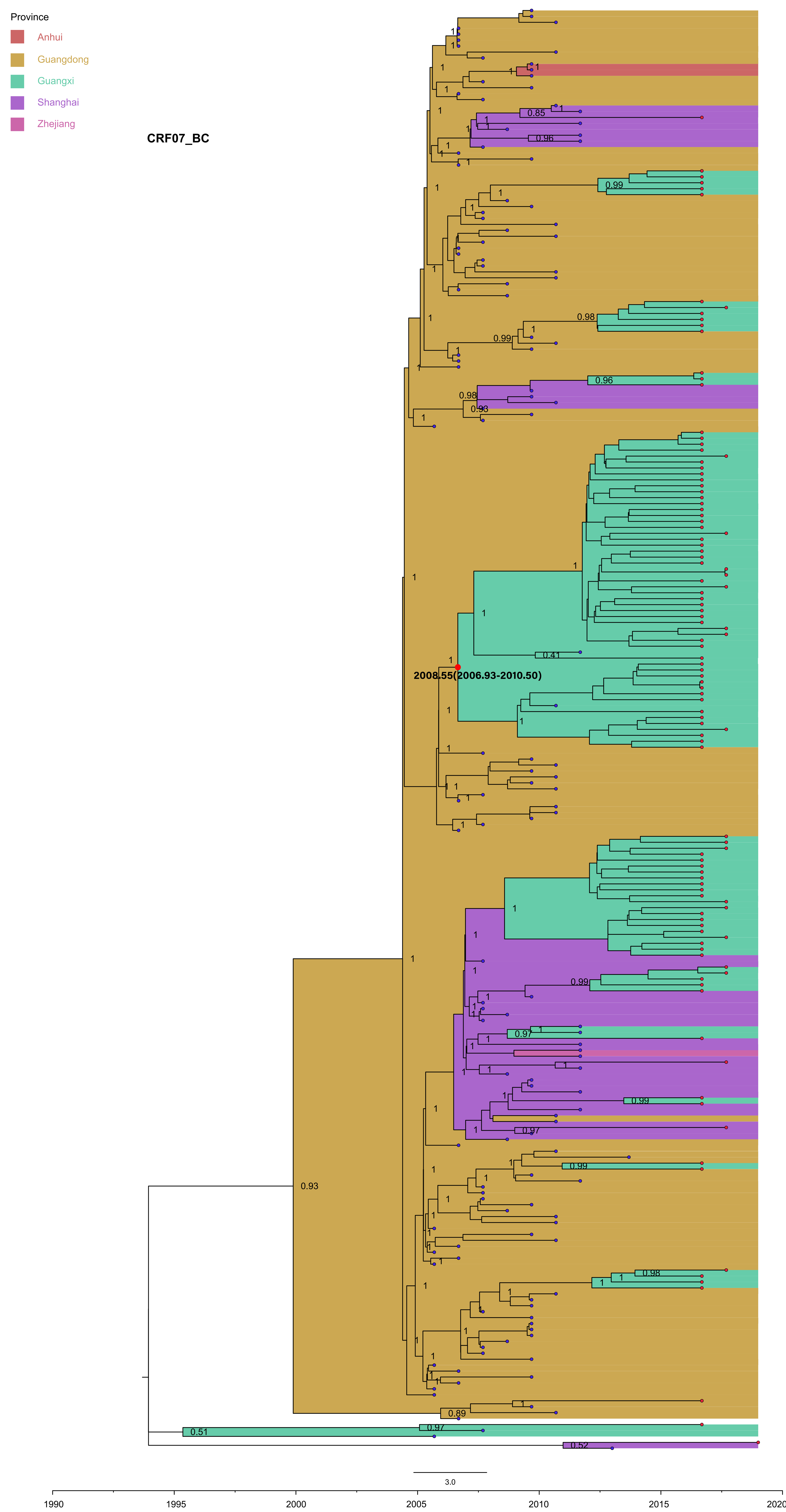

Province
Anhui
Guangdong
Guangxi
Shanghai
Zhejiang
CRF07_BC
2008.55(2006.93-2010.50)
3.0
1990
1995
2000
2005
2010
2015
2020

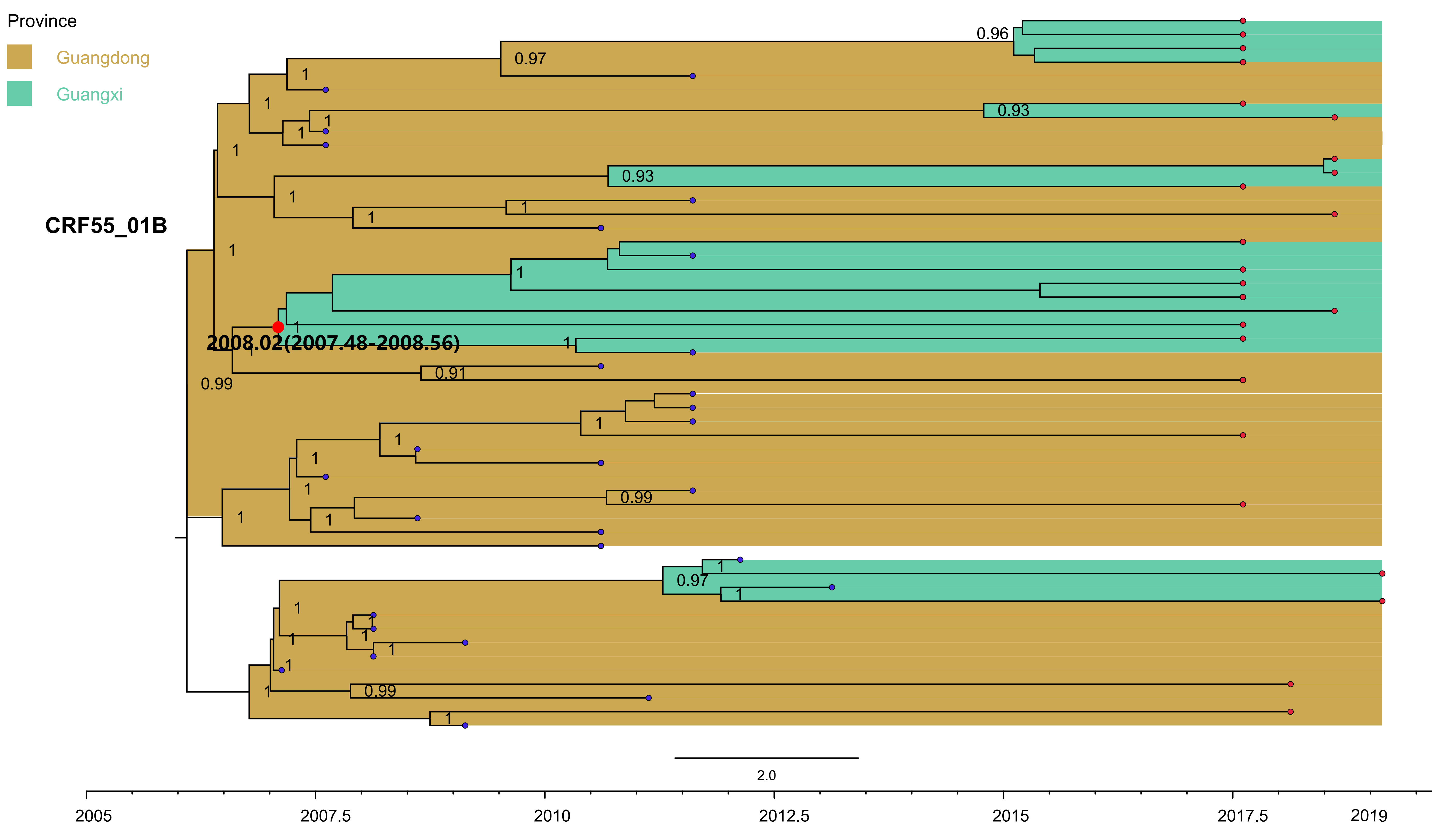
Province
Guangdong
Guangxi
CRF55_01B
2008.02(2007.48-2008.56)
0.96
0.97
0.93
0.93
0.91
0.99
0.99
0.97
0.99
2.0
2005
2007.5
2010
2012.5
2015
2017.5
2019

Figure S3. This figure illustrated time-scaled phylogeographic history of three HIV-1 subtypes (CRF01_AE, CRF07_BC, CRF55_01B) for MSM in Nanning, Guangxi. Background colors around branches represented the most probable origin province of the parental node of each branch. The names of the origin provinces were indicated by the color of the legend in the upper left corner. The values of probability inferred by Bayesian skygrid demographic model estimation for the most probable ancestral location were specified at the corresponding nodes. The probable time of introduction for these three key subtypes with effective population size circulating in Guangxi MSM was indicated at key nodes. Red and blue dots at the ends of branches denoted sequences of study participants and references, respectively. The Bayesian MCC trees were visualized through the FigTree software v1.4.3 (http://beast.bio.ed.ac.uk). The figure editing was using Adobe Illustrator 2020(V 24.0.1.341).
